# Biodegradable Electret Filters Based on Beeswax-Modified Fibers: A Novel Production Strategy

**DOI:** 10.3390/polym17060813

**Published:** 2025-03-20

**Authors:** Agata Penconek, Łukasz Werner, Zuzanna Bojarska, Arkadiusz Moskal

**Affiliations:** Faculty of Chemical and Process Engineering, Warsaw University of Technology, 00-645 Warsaw, Poland; zuzanna.bojarska@pw.edu.pl (Z.B.); arkadiusz.moskal@pw.edu.pl (A.M.)

**Keywords:** beeswax, electret filters, melt-blowing, nanoparticle filtration, PLA, solution-blowing

## Abstract

This research aims to create a high-efficiency, low-resistance biodegradable air-filter structure containing beeswax as a result of the simultaneous production of fibers by solution-blowing and melt-blowing. The melt-blowing method is effective for producing micrometer fibers on an industrial scale. In turn, the solution-blowing method allows for the production of fibers with a nanometric diameter from solutions containing temperature-sensitive additives such as beeswax. Combining these two methods is a promising perspective for producing high-performance, functional air-filter materials. Beeswax is a natural material capable of accumulating an electrical charge. When an external electric field is applied, the presence of beeswax in the filter structure facilitates charge retention on the fiber surface. This results in a fully biodegradable filter material with high efficiency and low resistance.

## 1. Introduction

Air filters should ensure high filtration efficiency in the broadest possible range of particle diameters. HEPA (High-Efficiency Particulate Air) or ULPA (Ultra-Low Penetration Air) filters provide high filtration efficiencies of 99.97% and 99.999%, respectively, but are also characterized by high pressure drops. Moreover, such filters are often made of petroleum-based materials, such as polypropylene or polyethylene, the production and disposal of which have a significant impact on the natural environment [1]. Commercially available filters are most often produced by melt-blowing techniques. This method is efficient and low-cost but requires polymers resistant to high temperatures, which are most often petroleum-based. In recent years, there has been growing interest in methods of producing filters from natural or biodegradable materials. For example, Atalia et al. used natural basalt fiber and cellulose fibers to produce a filter via an electrospinning process. Depending on the concentrations of ingredients, the filters achieved a filtration efficiency ranging from 87.93% to 99.99%, with a pressure drop of between 184 and 475 Pa [2]. In turn, Zhang et al. also produced fibers via electrospinning but using biodegradable polyvinyl alcohol (PVA) with cellulose fibers. The obtained filters can be characterized by a filtration efficiency for PM2.5 particles of up to 99.1% and a pressure drop of 91 Pa [3]. As can be seen, electrospinning is an effective method to produce nanometer-sized fibers from many natural polymers, such as chitosan, zein, or pea protein [4,5,6]. However, the technique’s efficiency is low due to low polymer flow rates. The third technique for producing fibers is solution-blowing. Solution-blowing is a safer method for creating fibers from both natural polymers, such as soy protein [7,8], as well as from synthetic but biodegradable ones, such as polylactic acid (PLA), poly-ε-caprolactone (PCL), or PVA [9,10,11]. Nonwoven filters manufactured using the solution-blowing method have a filtration efficiency of 96% for a wide range of particle sizes, with a pressure drop not exceeding 120 Pa [12].

Filtration efficiency is determined by four mechanisms of particle deposition on the fiber surface. Large particles are deposited on the fiber due to inertial impaction and interception, while small particles are deposited via diffusion. The fourth mechanism involves the interaction of external forces (gravitational forces for large particles and electrostatic interactions for submicron particles). Filtration efficiency will increase with a decrease in the average fiber diameter, an increase in the thickness of the filtration layer, or a reduction in porosity. However, an increase in filtration efficiency also leads to higher flow resistance. To improve filtration efficiency without increasing flow resistance, nanofibers can be incorporated into filters [13] or the filters can be designed as a layered structure [1]. Increasing filtration efficiency is particularly important for the most penetrating particle size (MPPS), i.e., particles in the 200–300 nm range. MPPS particles, due to their size, are not retained by inertial impaction and interception mechanisms. Particles of this size are deposited in the lungs’ alveoli, from where they are distributed throughout the body through the bloodstream, increasing the risk of asthma, heart disease, and lung and blood cancers [2].

One of the methods to increase the filtration efficiency of MPPS particles is the use of electrostatic interactions. The main methods for producing electrets are corona, thermo, induction and tribocharging [14,15]. Electrets capture neutral particles due to polarization effects that occur in the electret in a non-uniform electric field. In turn, charged particles are captured as a result of Coulombic interactions [15]. Both effects are stronger when the average fiber diameter is smaller [14]. Electrets achieve an initial filtration efficiency at the level of HEPA filters but with a much lower pressure drop. The high initial filtration efficiency is significantly reduced, up to half, over the time of the filter operation [16]. Three mechanisms can be involved: neutralization of the charge on the filter, screening of the filter charge, and chemical interactions between the aerosol and the material of the fibers [17]. One of the methods for slowing down the discharge of the filter is to incorporate dielectrics into the filter structure. The most popular dielectrics are SiO_2_ and BaTiO_3_ [18,19]. In turn, the first historically natural addition that allowed for the preservation of charge in the structure was a mixture of carnauba wax, resin, and beeswax [20]. Beeswax also exhibits bacteriostatic effects against such microbials as *Staphylococcus aureus*, *Salmonella enterica*, *Candida albicans*, and *Aspergillus niger* [21]. Therefore, its use during the production of filters can not only help preserve the charge but also give the filter additional properties, e.g., bacteriostatic ones.

Our research aimed to produce a high-efficiency electret by combining two techniques: melt-blowing and solution-blowing. Melt-blowing has been used as a highly efficient technique but is limited by its use of high-temperature-sensitive additives. Consequently, solution-blowing has been used as a technique by which nanometric fibers containing various additives can be obtained. Our previous research has shown that obtaining a filtration structure with good efficiency and lifetime is possible, depending on how these two techniques are combined. In both methods, we used biodegradable PLA polymer enriched with beeswax for solution-blowing. The fibers obtained in solution-blowing were added during the production stage. This allowed us to obtain a high-efficiency, biodegradable, nonwoven filter with a low pressure drop and high quality factor.

## 2. Materials and Methods

### 2.1. Solution-Blowing Spinning (SBS)

In the polymer solution-blowing process, a 10% (*w*/*w*) solution of polylactic acid (PLA) (Ingeo™ 6202D; NatureWorks^®^ LLC, Plymouth, MN, USA) in a chloroform/acetone (3/1) mixture (Sigma Aldrich, Poznań, Poland) was used. The PLA was placed in a chloroform/acetone mixture in a tightly closed container for 24 h at room temperature on a magnetic stirrer.

A 3%, 6%, and 12% (*w*/*w*) beeswax (100% natural, pure without additives) (Pallas, Bielsko-Biała, Poland) solution in 10% PLA in a chloroform/acetone (Sigma Aldrich, Poznań, Poland) mixture was used in the research. The beeswax and PLA were placed in a mixture of chloroform/acetone (3/1) in a tightly closed container on a magnetic stirrer for 20 min in a water bath at 60 °C to dissolve the beeswax. After this time, the sample was transferred to room temperature and left for 24 h on a magnetic stirrer.

Solution-blowing was carried out in a system consisting of 4 nozzles with an internal diameter of 1 mm and an external diameter of 5 mm, powered by a polymer dosed using a precision syringe pump (Legato270; KDScientific, Holliston, MN, USA) at a flow rate of 2 mL/min (polymer flow through a single nozzle was 0.5 mL/min). Compressed air was supplied to the system at a flow rate of 400 L/min (airflow through one nozzle was 100 L/min). The experimental set-up is described in detail in our previous publication [22].

**Figure 1:**
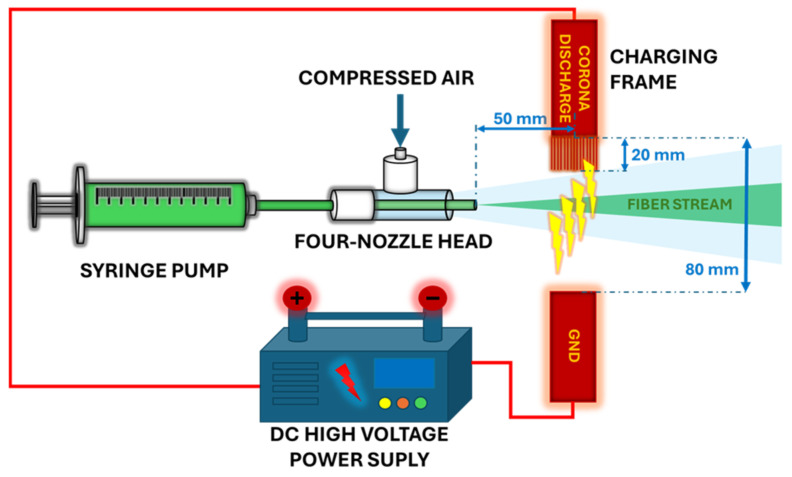
The solution-blowing installation scheme.

The charging process was carried out by using the charging frame situated 5 cm in front of the four nozzles (Figure 1). In the charging frame (120 mm × 80 mm), 80 stainless needles with a diameter of 0.45 mm and 20 mm length were connected to a DC high-voltage supply providing 12 kV and 15 kV voltages and 80 microamps. The system provided an electric field of 503 and 948 V/m for the 12 kV and 15 kV voltages, respectively (measured at the polymer outlet from the nozzles). The electric field was measured using a VT-ER electromagnetic radiation tester (Beijing, China).

### 2.2. Melt-Blowing (MB)

**Figure 2:**
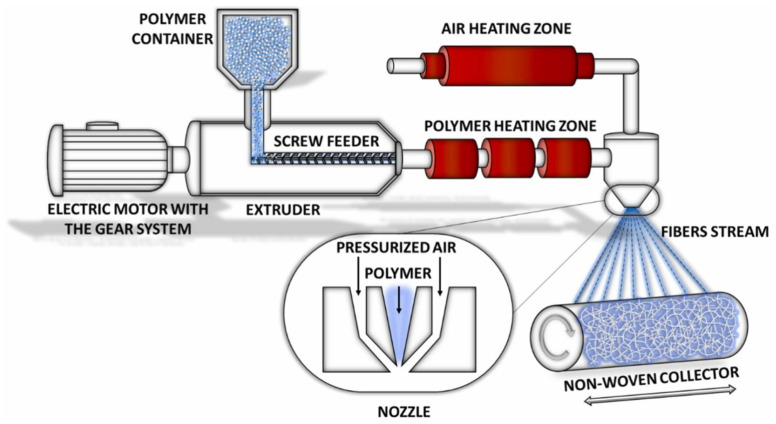
The scheme for the melt-blown system for the production of nonwoven materials. Reproduced with permission [23]. Copyright © 2016 Elsevier B.V.

The main fibrous framework of all the manufactured materials was produced using melt-blowing (MB) technology. The scheme of the MB system is presented in Figure 2. In this process, PLA granules are melted and heated to 210 °C by the heating system. The molten polymer is then transported to the nozzle, where it meets a stream of hot air (210 °C). By adjusting key process parameters—including the flow rates of polymer and air, temperature in the heating system and the nozzle, as well as the collector’s rotation speed and its distance from the nozzle, it is possible to obtain nonwoven materials with a defined structure and morphology (more for a detailed description, see [22]).

Polylactic acid (NatureWorks^®^ LLC Ingeo™ 3251D Polylactic Acid; Plymouth, MN, USA) is designed for injection molding applications. However, it can also be used for fiberization in the MB process due to its higher melt flow capability compared with other resin grades currently available on the marketplace. The typical properties of this include a melt flow index (MFI) of 80 g/10 min (measured at 210 °C/2.16 kg) and a density of 1.24 g/cm^3^ at 23 °C.

### 2.3. Combining Solution-Blowing and Melt-Blowing

The melt-blowing nozzle with the flowing molten polymer is placed above the rotating collector. Polymer flows from the solution-blowing nozzle are generated by a syringe pump, so the nozzle (and the entire solution-blowing system) is positioned in front of the collector. The distance of the melt-blowing and solution-blowing nozzles from the collector was 35 cm and 37 cm, respectively. The research analyzed three variants:

v1: Filters produced only by melt-blowing from PLA;

v2: Filters produced simultaneously using melt-blowing and solution-blowing techniques from a PLA solution;

v3: Filters produced simultaneously using melt-blowing and solution-blowing techniques from a PLA solution containing beeswax at a concentration of 3%, 6%, and 12% (*w*/*w*).

Additionally, for the v2 and v3 variants, corona charging at 12 kV and 15 kV voltages and 80 microamps were also used. The polymer fiberization time was 8.5 min for each trial.

The mass of fibers produced during solution-blowing was determined based on the test in which only fibers from the solution-blowing nozzle were deposited onto the collector. At least three filter fabrics were manufactured for each variant of the experiment.

### 2.4. Fiber Characterization

The morphology of the filtration structures was determined based on SEM images (TM-1000; Hitachi, Tokyo, Japan). The fibers were sputtered with a 25 nm layer of gold before being subjected to SEM analysis (K550X EMITECH Quorum; Laughton, East Sussex, UK).

Fourier-transform infrared spectroscopy (FTIR) analysis was performed on unmodified nonwoven material, nonwoven material modified with wax additives, and pure beeswax using a Nicolet iS10 spectrometer (Thermo Scientific, Waltham, MA, USA) in attenuated total reflection (ATR) mode with a diamond crystal. Measurements were conducted in the range of 500–4000 cm^−1^ with a resolution of 4 cm^−1^. FTIR analysis was utilized to verify the presence of wax on the fibers by identifying their characteristic functional groups. The obtained spectra allowed us to confirm the successful incorporation of wax into the fiber structure.

### 2.5. Filtration Efficiency and Pressure Drop

**Figure 3:**
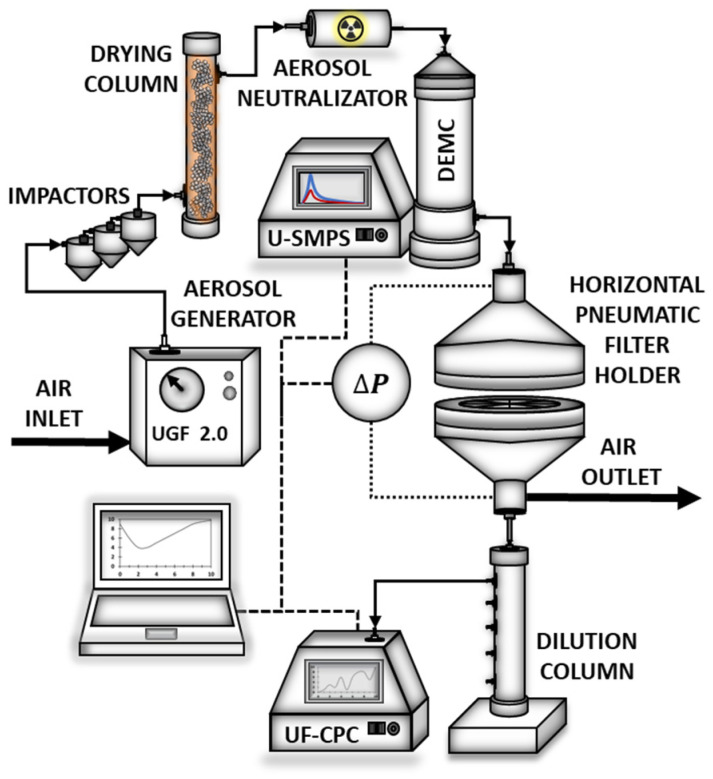
The scheme of the MFP Nano plus testing rig used for the filtration nanodroplet test. Reproduced with permission [23]. Copyright © 2016 Elsevier B.V.

The modular MFP Nano plus test bench (PALAS GmbH, Karlsruhe, Germany) was used to perform aerosol filtration experiments on the manufactured materials. The scheme of the apparatus is presented in Figure 3. The aerosol was generated from 0.03% di-ethyl-hexyl-sebacat (DEHS oil) (Sigma-Aldrich, Poznań, Poland) diluted in isopropanol (Sigma-Aldrich, Poznań, Poland). Using the UGF 2000 liquid nebulizer (Karlsruhe, Germany), which is equipped with a binary nozzle and cyclone, it is possible to generate droplets within a diameter range from 20 to 200 nm. The droplets produced by the UGF nebulizer are directed toward impactors, where the largest droplets are removed from the gas stream. Then, in the Kr-85 bipolar neutralizer, the carrier gas is ionized, and the aerosol particles attain a defined equilibrium charge distribution. Next, in the DEMC classification column, droplets are classified based on their electrical mobility, which depends on their size. After classification, the aerosol is introduced into the filtration test chamber, where the pressure difference across the tested material (pressure drop) is measured at a constant flow rate. From the filter holder chamber, after passing through the dilution columns, the concentration of the aerosol droplets is determined in the universal fluid condensation particle counter (UF-CPC).

**Figure 4:**
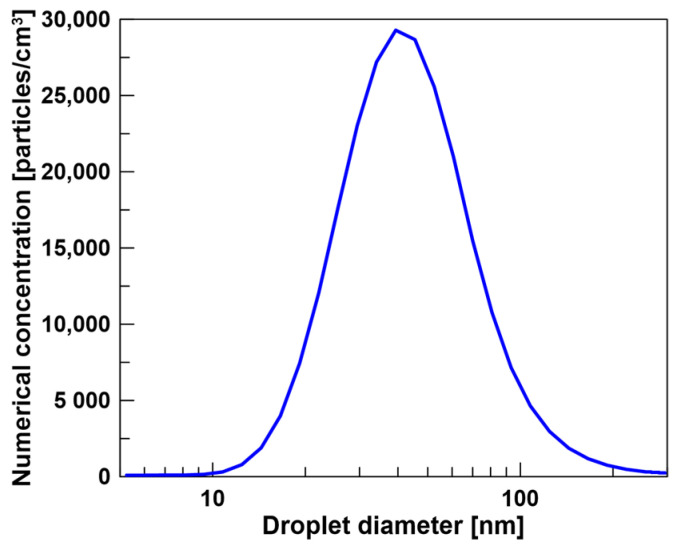
Numerical concentration size distributions of the tested DEHS oil droplet aerosols in the range 20–200 nm.

The working area of the filtration material was 0.01 m^2^. All aerosol separation measurements were conducted at an air-face velocity of 0.158 m/s and at a flow rate of 95 L/min. The average numerical concentration of DEHS droplets was 2.46∙× 10^5^ particles/cm^3^. The size distribution of the generated oil droplets used in the experiments is presented in Figure 4.

Filtration efficiency tests were conducted for all the material variants (MB, MB-SBS, MB-SBS wax 3%, MB-SBS wax 6%, and MB-SBS wax 12%) in both raw and electrically charged conditions. The experiments followed a measurement sequence consisting of an initial assessment without a filter (upstream) and a subsequent assessment with a filter (downstream) in the tested chamber. This procedure was required since the filtration system is equipped with only a single suction probe, which draws the aerosol into the counter. For each material variant, three alternating upstream and downstream measurements were performed. The obtained results were averaged.

### 2.6. Quality Factor

An important parameter enabling filter quality assessment in terms of filtration efficiency and pressure drop is the quality factor (Q_F_). The Q_F_ was determined from the following relation:

$$Q_{F}=\frac{-ln\left(1-\frac{E}{100}\right)}{∆P}$$

where E is the filtration efficiency (%) and ΔP is the pressure drop (in Pa).

The quality factor is a parameter that combines two critical characteristics of filters from a filtration perspective: filtration efficiency and pressure drop. The higher the filtration efficiency and the lower the pressure drop, the greater the quality factor. Filtration efficiency can be increased, for example, by reducing the average fiber diameter, decreasing the porosity, or increasing the thickness. However, these changes also lead to a higher pressure drop.

## 3. Results and Discussion

### 3.1. Morphology of Fibers

**Figure 5:**
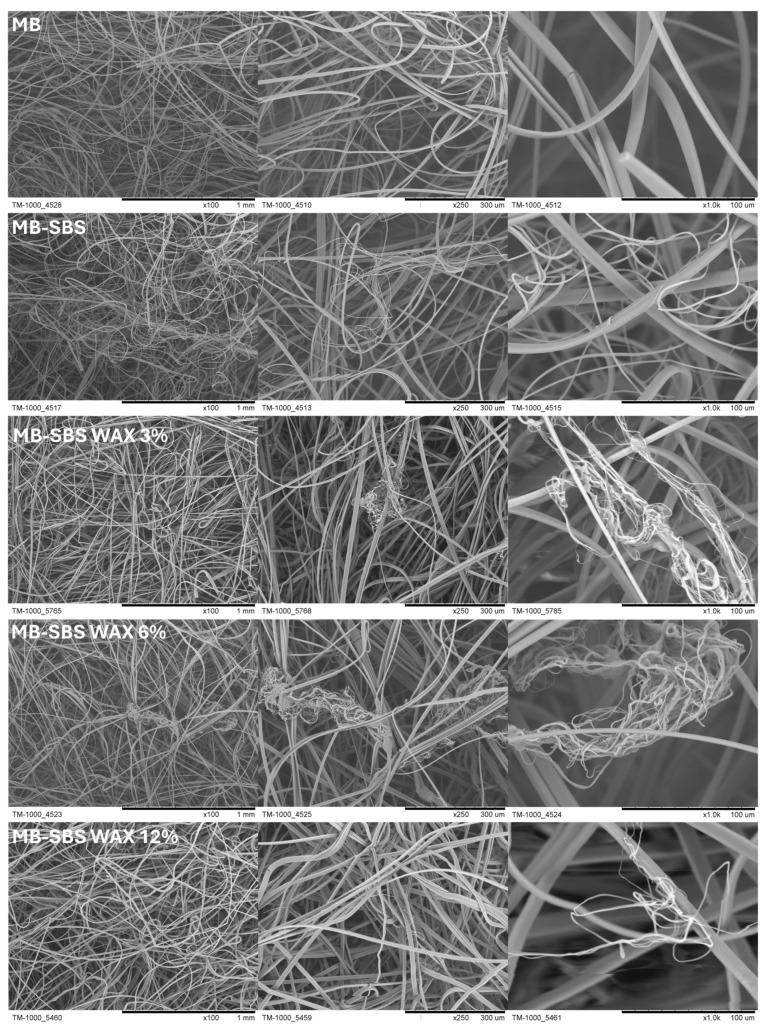
SEM images of nonwoven fabrics showing various morphologies of the manufactured filters.

Figure 5 shows PLA fibers produced using the melt-blowing technique, as well as from combining the melt-blowing and solution-blowing techniques and by using a mixture of PLA and beeswax in various concentrations. The obtained fibers have a smooth surface. The fibers created with the MB technique have larger diameters and are in greater quantities than the finer-diameter fibers created with the SBS technique. In the SBS technique, fibers are created due to the impact of air expanding to atmospheric pressure on the polymer solution flowing out of the nozzle. Under the influence of shear forces caused by the flowing air, polymer drops change their shape to a conical one. The fiber is created when the shear force exceeds the interfacial tension at the polymer–air boundary. On the way from the nozzle to the collector, the solvent evaporates, and, as a result, the formed fiber reaches the collector. This fiber formation mechanism favors the formation of fibers with a submicron diameter.

Mass measurements of the obtained structures confirm these observations. The average mass of the filter created by combining the MB and SBS techniques is 66.25 ± 2.97 g, while the average mass of fibers produced using the SBS technique is 0.33 ± 0.11 g; the mass of SBS fibers is, on average, 0.5% of the mass of the entire filter.

**Figure 6:**
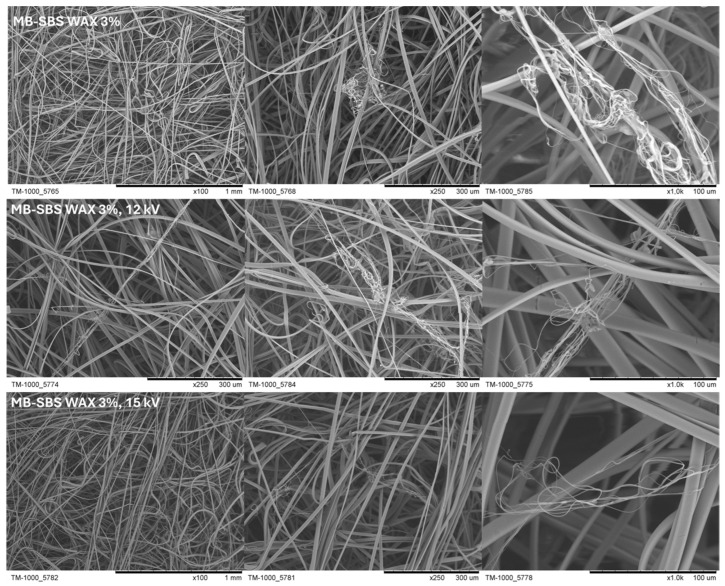
SEM images of nonwoven fabrics showing various morphologies of the fibers containing beeswax charged in an external electric field.

Based on the SEM images of fibers made from a combination of MB and SBS with 3% wax (Figure 6), it was shown that the use of an external field to charge the fibers during their production using the SBS method (regardless of the voltage used) did not affect the appearance of the fibers. The fibers still had a smooth surface and a smaller diameter than those produced using the MB technique.

Regarding electret filters, it is important to note that storage conditions play a critical role in determining the stability of electrostatic charges on the fibers and, consequently, the filtration efficiency of electret materials. Specifically, elevated temperature and humidity accelerate the dissipation of electrostatic charges. The magnitude of this effect is highly dependent on the material’s intrinsic properties, such as its dielectric constant, surface energy, and wettability.

Studies [24,25] have shown that exposing filtration materials to high temperatures can increase the diffusion of electrostatic charges, leading to a significant reduction or even complete neutralization of these charges. Likewise, elevated humidity levels can accelerate the electrostatic charge dissipation. The presence of moisture facilitates the adsorption of water molecules onto the surface of the filter fibers, leading to charge screening and subsequent neutralization. Other investigations show that external (e.g., electron beams) irradiation that can be used for mask disinfection will also significantly affect the reduction in the filtration efficiency of materials [26]. Nevertheless, the electret filters stored under neutral environmental conditions can maintain charge stability on the fibers, thereby ensuring high filtration efficiency [27].

### 3.2. FTIR Analysis

**Figure 7:**
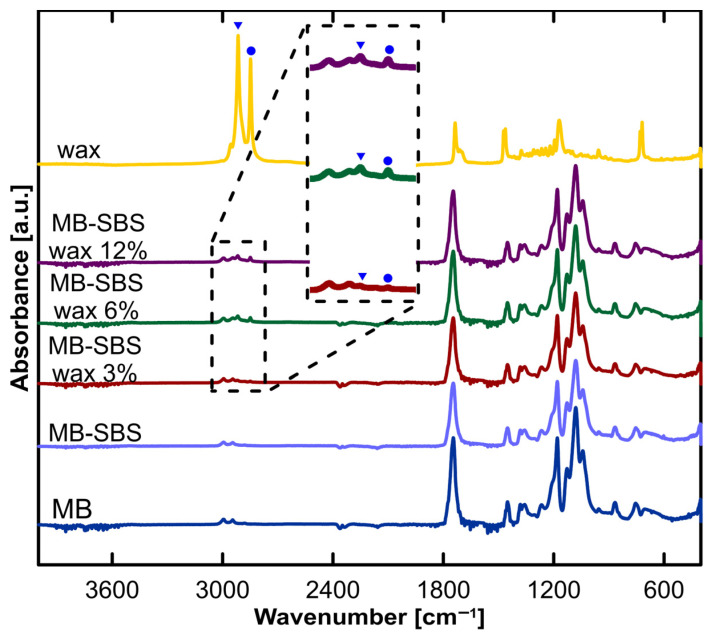
FTIR spectra of nonwoven fabrics prepared using the melt-blowing technique (MB), filters prepared by melt-blowing and solution-blowing (MB-SBS), materials modified with beeswax additives (MB-SBS-wax), and pure beeswax. Peaks corresponding to the symmetric stretching of aliphatic hydrocarbons from beeswax at 2915 cm^−1^ (▼) and 2850 cm^−1^ (●).

The FTIR spectra of the nonwoven materials MB and MB-SBS (Figure 7) showed typical stretching frequencies for PLA, specifically for C–O and C=O, at 1080 cm^−1^ and 1750 cm^−1^, respectively. Additionally, characteristic bending frequencies for asymmetric and symmetric -CH_3_ vibrations were observed at 1455, 1363, 2945, and 2995 cm^−1^ [28]. Modified materials with beeswax additives exhibited additional weak peaks corresponding to the symmetric stretching of aliphatic hydrocarbons from wax at 2915 cm^−1^ (▼) and 2850 cm^−1^ (●). The FTIR spectrum of wax also displayed bands corresponding to vibrations of aliphatic hydrocarbons at 720 cm^−1^ and 1470 cm^−1^. In the case of beeswax, additional bands appeared at 1739 cm^−1^ and 1175 cm^−1^, which can be attributed to the carboxyl groups of fatty acids and esters [29].

### 3.3. Filtration Efficiency

**Figure 8:**
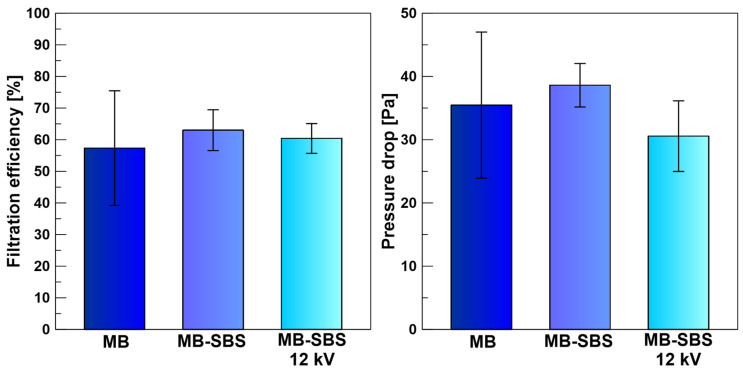
Filtration efficiency and pressure drop for PLA: MB, MB-SBS, and MB-SBS 12 kV filters.

High filtration efficiency with low pressure drop is a desirable feature when designing filters. Higher filtration efficiency can be achieved by incorporating nanofibers into the filtration structure. Due to their large surface, nanofibers can retain more particles than micrometer fibers. However, too many nanofibers will cause a significant increase in airflow resistance, i.e., a large pressure drop. As our results show, with a 0.5% share of nanofibers in the MB-SBS filter, an insignificant increase in filtration efficiency and pressure drop can be observed in comparison with the MB filter (Figure 8). Although theoretical considerations, which can be found in Przekop and Gradaoń’s work [13], suggest that a small portion of nanofibers in the filtration structure is enough to significantly increase the filtration efficiency without increasing the pressure drop.

The second way to increase the filtration efficiency, especially for nanoparticles, is to implement an electric charge on the filter surface. Due to an electric charge, electrostatic interactions attract charged particles, thereby increasing deposition inside the filter and improving filtration efficiency without causing an increase in the pressure drop. However, our research did not observe an increase in the filtration efficiency of MB-SBS filters in which fibers made by the SBS technique were charged in an external electric field (12 kV) (Figure 8). The reasons for this are the insufficient number of charged fibers and the poor dielectric properties of PLA [30].

### 3.4. The Influence of Beeswax Concentration on Filtration Efficiency

**Figure 9:**
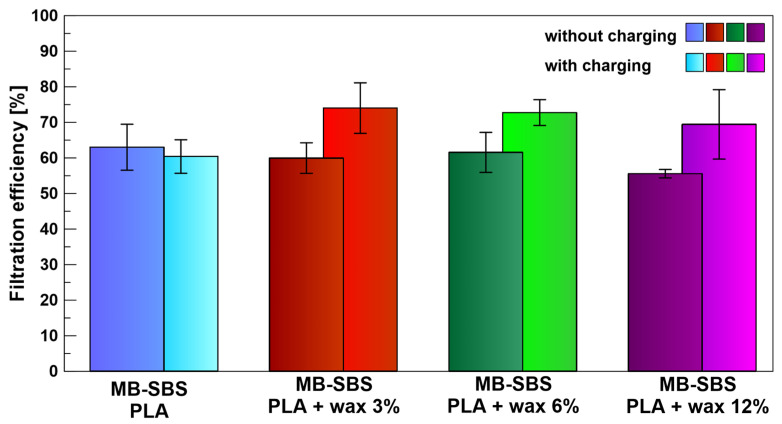
The filtration efficiency of MB-SBS filters charged with a 12 kV corona-charging voltage and MB-SBS materials containing beeswax in different concentrations.

In the case of materials that were not subjected to electrostatic charging, the presence of SBS fibers (both with and without wax) had no significant impact on the filtration efficiency. These fibers constitute only a small proportion of the filtration structure (0.5%), and their presence is not significant enough to noticeably increase the ability and available surface area for particle capture. The results presented in Figure 9 indicate that their presence (regardless of the wax content) does not significantly affect the filtration efficiency. The observed differences fall within the margin of statistical error. Beeswax present in the fibers, if not exposed to an electric field, can even cause a slight reduction in the filtration efficiency. This is most likely due to the increase in the average diameter of the fibers created using the SBS technique, as the concentration of beeswax in the PLA/beeswax mixture increases, which was observed in previous studies [31].

Beeswax, being a natural dielectric material, can improve the poor dielectric properties of PLA. Moreover, thanks to its natural origin, it does not affect the biodegradability or environmental friendliness of the produced filter, and it has properties that enable it to accumulate an electric charge. The results clearly indicate a synergistic effect between the presence of wax and the charging that occurs in an external electric field (Figure 9). The most significant increase in efficiency was observed for a 3% addition of beeswax (11 percentage points compared with the mixture of MB-SBS fibers not charged in an electric field) and for a 6% addition of beeswax (10 percentage points). At the same time, no significant changes in the pressure drop were observed during the airflow through the tested filter (Table 1).

### 3.5. The Influence of the Voltage of Corona Charging on Filtration Efficiency

**Figure 10:**
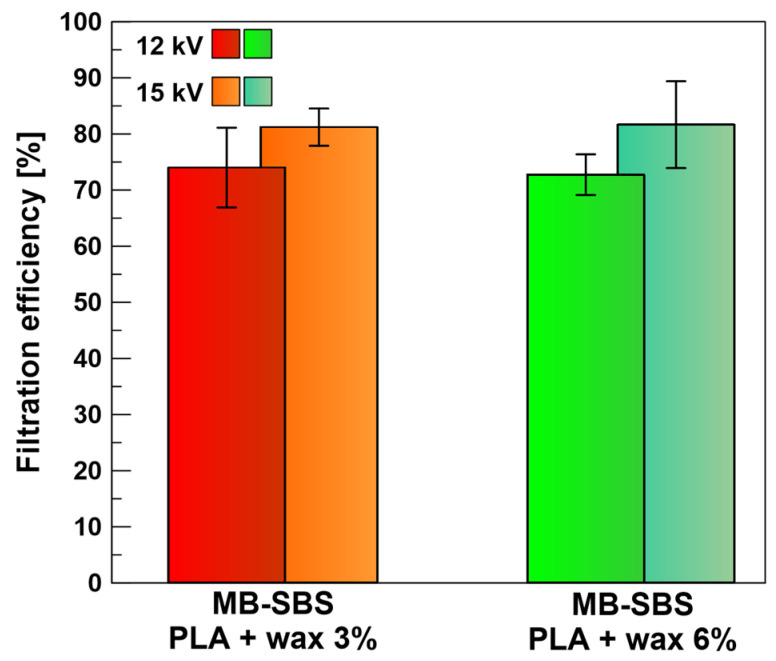
The filtration efficiency of MB-SBS filters charged at a 12 and 15 kV corona-charging voltages containing beeswax in different concentrations.

The use of a mixture of PLA and beeswax and an external electric field (corona discharge) during the production of SBS fibers resulted in the produced filters becoming electrets, i.e., materials that generate a quasi-steady external electric field under the influence of polarization. The charging of the nonwoven fabric introduced an additional deposition mechanism based on electrostatic interactions (Coulombic, image, and polarization forces), resulting in a more effective capture of particles from the aerosol stream [32,33,34]. Chen et al. [35] showed that the filtration efficiency of melt-blowing polypropylene electret filter increases as the voltage of corona charging increases. Therefore, we analyzed the influence of the voltage of corona charging in producing fibers using the solution-blowing method on the filtration efficiency of MB-SBS filters containing beeswax. Concentrations of 3% and 6% beeswax were selected for further analysis (MB-SBS 3% wax and MB-SBS 6% wax filters in an external electric field showed higher filtration efficiency than the MB-SBS 12% wax filter). Figure 10 presents the filtration efficiencies of MB-SBS filters containing beeswax obtained at corona charging voltages of 12 and 15 kV. A higher voltage generates a stronger electric field, causing more intense ionization of air molecules, which leads to a greater number of free ions that can be deposited onto the fibers. Additionally, at a higher voltage, in addition to the deposition of charges on the surface, charge trapping may also occur within the material’s structure, leading to a higher charge accumulation on the fibers [36]. An increase in the corona-charging voltage enhances the filtration efficiency, similar to what was observed for polypropylene filters produced by the melt-blowing method [35]. It is worth noting that the impact of voltage also depends on the composition of the filtration material, including its dielectric constant. In the case of fibers that do not contain beeswax, despite the charging process, no increase in efficiency is observed, which suggests a reduced ability of the material to accumulate electrostatic charge on its surface. With a higher voltage of corona-charging, an average increase of 3.5 Pa in pressure drop is observed, increasing to 35.18 ± 7.63 Pa and 30.24 ± 7.58 Pa for 3% and 6% wax additives, respectively.

**Figure 11:**
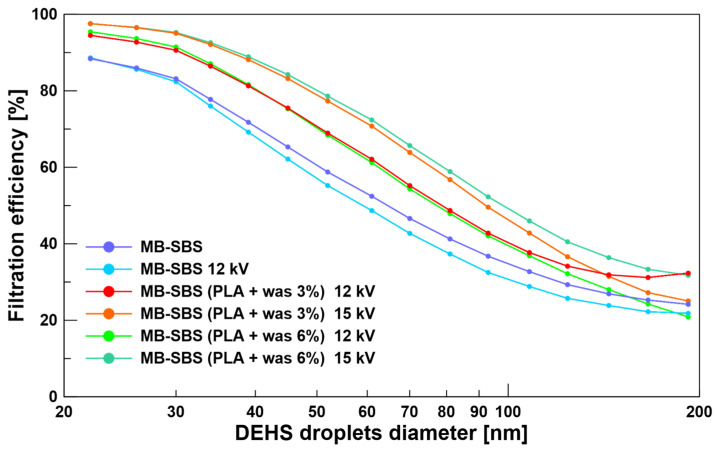
Fractional filtration efficiency for MB-SBS filters without beeswax and those containing 3% and 6% beeswax additives charged at 12 and 15 kV corona-charging voltages.

Several deposition mechanisms are responsible for the capture of particles and droplets on fibers, including mechanical mechanisms (interception, inertial impaction, gravitational settling, and diffusion) as well as electrostatic interactions [37,38]. Depending on the droplet diameter, these mechanisms play different roles and dominate in different size ranges. Electrostatic forces and diffusion, due to the small size of nanoparticles, can easily influence their trajectory. As the particle diameter increases, both mechanisms lose significance, and the capture efficiency approaches a point where neither mechanism is dominant—the particles are too small for mechanical mechanisms to have a significant impact [39]. This trend can be observed in the fractional efficiency graph (see Figure 11), which presents the fractional filtration efficiency of MB-SBS filters without beeswax or with 3% and 6% beeswax additives charged at a 12 or 15 kV corona voltage. The effectiveness of the process decreases as the diameter of the filtered droplets increases. Nevertheless, in charged materials, the efficiency is enhanced significantly for droplets up to 100 nm.

### 3.6. Quality Factor

**Figure 12:**
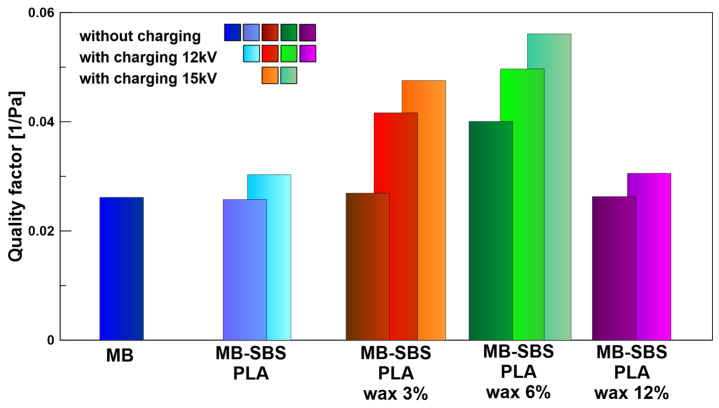
Initial filter quality factor Q_F_ (1/Pa) of MB-SBS nonwoven fabrics, uncharged and charged with 12 and 15 kV corona-charging voltages, and materials containing beeswax in different concentrations.

Figure 12 shows the quality factor for all the produced filters. The highest Q_F_ value of 0.0561 1/Pa was obtained for the MB-SBS filter containing 6% beeswax charged with a 15 kV corona-charging voltage. The obtained Q_F_ value exceeds the Q_F_ (0.046 1/Pa) obtained for polypropylene filters produced by other authors [40] using the melt-blowing technique and corona charging. The obtained Q_F_ value is also an order of magnitude higher than the Q_F_ obtained in our previous research (0.008 1/Pa), in which filters were also produced using a combination of melt-blowing and solution-blowing techniques. However, polypropylene was used for melt-blowing and PLA for solution-blowing [22]. Since the filter is made of a biodegradable PLA polymer containing natural beeswax, the results are promising for the development of high-efficiency filter materials with low environmental impact.

## 4. Conclusions

The presented research has shown that it is possible to produce biodegradable, high-efficiency filters with a low pressure drop and a high quality factor value. Combining two fiber production techniques, melt-blowing and solution-blowing, supports the design of a filter with unique properties. Fibers made using the MB technique ensure the appropriate strength and weight of the filter. Fibers created using the SBS technique increase the filtration efficiency thanks to their fine size and allow for the incorporation of compounds into the entire filter that could not be used in the MB technique due to the high temperature required for polymer melting. In the presented research, fibers using the SBS technique were made from a mixture of PLA and beeswax, which improved the dielectric properties of the filter and enabled the creation of a filter with a high quality factor. Beeswax may also have bacteriostatic properties, which would also be a highly desirable feature in the case of filters.

## Figures and Tables

**Table 1:** Pressure drop of MB-SBS filters obtained in an electrical field (12 kV) containing beeswax in different concentration (SD—standard deviation).

	Pressure Drop [Pa]			
	Without Charging		With Charging	
	Mean	SD	Mean	SD
MB-SBS (PLA)	38.61	3.44	30.56	5.58
MB-SBS (PLA + wax 3%)	34.00	7.29	32.36	6.61
MB-SBS (PLA + wax 6%)	23.86	2.62	26.17	3.86
MB-SBS (PLA + wax 12%)	30.86	6.83	38.80	11.74

## Data Availability

The original contributions presented in this study are included in the article. Further inquiries can be directed to the corresponding author(s).
